# Supplementary material for: China’s Trade of Agricultural Products Drives Substantial Greenhouse Gas Emissions
Source: Int J Environ Res Public Health. 2022 Nov 27;19(23):15774. doi: 10.3390/ijerph192315774 (PMC9740673; doi:10.3390/ijerph192315774)
Supplement: Supplementary file 1 [file ijerph-19-15774-s001.zip › ijerph-2020763-supplementary.pdf]

Supplementary materials

Table S1. GHG emissions from agricultural activities.

Table S2. The classification details and codes of China's agricultural products.

Table S3. Embodied GHG emissions in the exports of China's agricultural products during 1995-2015.

Table S4. The proportion of embodied GHG emissions in the exports during 1995-2015.

Table S5. The embodied GHG emissions in the exports of China's agricultural products to major countries in 2015.

Table S6. The embodied GHG emissions in the imports of China's agricultural products during 1995-2015.

Table S7. The proportion of embodied GHG emissions in the imports during 1995-2015.

Table S8. The embodied GHG emissions in the imports of China's agricultural products from major countries in 2015.

Table S9. The variation trend of net import and export volume of China's agricultural products.

Table S10. The variation trend of embodied GHG emissions in the net import and export of China's agricultural products.

Table S11. The embodied GHG emissions in the net imports of China's

agricultural products with major countries from 1995 to 2015.

Table S12. The embodied GHG emissions in the net imports of China's agricultural products with major countries in 2015.

Table S13. The export volume of China's agricultural products from 1995 to 2015.

Table S14. The major export trading partners in 2015.

Table S15. The import volume of China's agricultural products from 1995 to 2015.

Table S16. The major import trading partners in 2015.

Table S17. Carbon emission factors (CEFs) of China's trading partners of agricultural products provided by FAO.

Table S1. GHG emissions from agricultural activities.

Production activities of crops	Rice planting(CH_4); Energy using(CH_4); Synthetic fertilizers(N_2O); Fertilizer applied to soil(N_2O); Dung left in pasture (N_2O); Crop residues (N_2O); Organic soil (N_2O); Burn as residue (N_2O、CH_4); burning savanna (N_2O、CH_4).
Production activities of livestock products	Intestinal fermentation (CH_4);<br>Fertilizer management (N_2O、CH_4)

Source：[1]

Table S2. The classification details and codes of China's agricultural products.

Item	Code	Name
Beef	02012000	Fresh and cold bone-in beef
	02013000	Fresh, cold boneless beef
	02021000	Frozen whole head and half head of beef
	02022000	Frozen bone-in beef
	02023000	Frozen boneless beef
	02062100	Frozen beef tongue
	02062200	Frozen beef liver
	02062900	Other frozen beef mince
	02102000	Dried, smoked, salted or salted beef
Pork	02031200	Fresh, cold bone-in pork foreleg, pork hind leg and its meat pieces
	02031900	Other fresh and cold pork
	02032110	Frozen whole head and half head of suckling pork
	02032190	Other frozen whole head and half head pork
	02032200	Frozen bone-in pork foreleg, pork hind leg and its meat pieces
	02032900	Other frozen pork
	02064100	Frozen pork liver
	02064900	Other frozen pork mince
	02101110	Dried, smoked, salted or salted bone-in pork shanks
	02101190	Dried, smoked, salted or salted other bone-in pork pieces
	02101200	Dried, smoked, salted or salted pork belly (pancetta)
	02101900	Other dried, smoked, salted or salted pork
Mutton	02042200	Fresh and cold bone-in lamb
	02043000	Frozen whole head and half head of lamb
	02044100	Frozen whole head and half head of lamb
	02044200	Frozen lamb with bones
	02044300	Frozen boneless lamb
	02045000	Goat meat
Poultry	02071100	Whole chicken, fresh or cold
	02071200	Whole chicken, frozen
	02071311	Bone-in chicken pieces, fresh or cold
	02071329	Other chicken mince, fresh or cold
	02071411	Frozen chicken pieces with bones
	02071419	Other frozen chicken pieces
	02071421	Frozen chicken wings (excluding wing tips)
	02071422	Frozen chicken claws
	02071429	Other frozen chicken morsels
	02072500	Whole turkey, frozen
	02072700	Turkey pieces and miscellaneous, frozen
	02074100	Whole duck, fresh or cold
	02074200	Whole frozen duck

	02074400	Duck pieces and miscellaneous, fresh or cold
	02074500	Frozen whole duck pieces and miscellaneous
	02075100	Whole goose, fresh or cold
	02075200	Whole frozen goose
	02075400	Fresh or cold goose pieces and miscellaneous
	02075500	Frozen goose pieces and miscellaneous
	02081020	Frozen rabbit meat, excluding rabbit head
	02089010	Fresh, cold, frozen squab meat and edible miscellaneous mince
	04011000	Unconcentrated and not sweetened with sugar or other sweetening substances milk and cream with a fat content of not more than 1%.
	04012000	Milk and cream not concentrated and not sweetened with sugar or other sweetening substances with a fat content of 1% <6%.
	04015000	Milk and cream, not concentrated and not sweetened with sugar or other sweetening substances, with a fat content of more than 10%
	04021000	Powdered, granulated or other solid milk and cream with a fat content ≤ 1.5
	04022100	Powdered, granulated or other solid milk and cream with more than 1.5% fat, not sweetened with sugar or other sweetening substances
	04022900	Powdered, granulated or other solid milk and cream, fat content > 1.5%, sweetened with sugar or other sweetening substances
	04029100	Non-solid milk and cream, sweetened with no sugar or other sweetening substances
Milk	04029900	Non-solid milk and cream, sweetened with sugar or other sweeteners
	04031000	Yogurt
	04039000	Other buttermilk, caked milk and cream, yogurt and other fermented or acidified milk and cream
	04041000	Whey and modified whey, whether or not concentrated, sweetened or otherwise
	04049000	Products containing natural dairy products, whether sweetened or unsweetened, not listed
	04051000	Butter
	04052000	Milk spreads
	04059000	Other fats and oils derived from milk
	04061000	Fresh cheese, including whey cheese; curds
	04062000	All kinds of grated or powdered cheeses
	04063000	Processed cheeses (except grated or powdered)
	04064000	Blue cheese and other textured cheeses produced by penicillium
	04069000	Cheeses not listed
	04071100	Fertilized eggs for hatching
	04072100	Fresh eggs
	04072900	Other fresh eggs
Egg	04079010	Salted eggs
	04079020	Dried eggs
	04079090	Unlisted pickled or boiled eggs in shell
	04081100	Dried egg yolk

	04081900	Other egg yolks
	04089100	Dry shelled eggs
	04089900	Other shelled eggs
Sugar	04090000	Natural honey
	04100010	Bird's nest
	04100041	Fresh Royal Jelly
	04100042	Fresh Royal Jelly powder
	04100043	Bee pollen
	04100049	Other bee products
Vegetable	07011000	Seed potatoes
	07019000	Fresh or frozen potatoes, except for seed
	07020000	Fresh or frozen tomatoes
	07031010	Fresh or frozen onions
	07031020	Fresh or frozen green onions
	07032010	Fresh or frozen garlic
	07032020	Fresh or frozen garlic moss and garlic plants (green garlic)
	07032090	Other fresh or frozen garlic
	07039010	Fresh or frozen leeks
	07039020	Fresh or frozen shallots
	07039090	Fresh or frozen other onion vegetables
	07041000	Fresh or frozen cauliflower and hard-flowered kale
	07042000	Fresh or frozen rosette kale
	07049010	Cabbage, fresh or frozen
	07049020	Other fresh or frozen broccoli
	07049090	Fresh or frozen edible mustard vegetables not listed
	07051100	Fresh or chilled bulb lettuce (lettuce with hearts)
	07051900	Other fresh or frozen romaine lettuce
	07052100	Vetrov chicory, fresh or chilled
	07052900	Other fresh or frozen chicory
	07070000	Cucumbers and gherkins, fresh or frozen
	07081000	Peas, fresh or frozen
	07082000	Cowpeas and beans, fresh or frozen
	07089000	Other fresh or frozen legumes
	07092000	Fresh or frozen asparagus
	07093000	Fresh or frozen eggplant
	07094000	Fresh or frozen celery (except tubers)
	07095100	Mushrooms of the genus Paramecium, fresh or frozen
	07095910	Fresh or frozen matsutake mushrooms
	07095920	Fresh or frozen shiitake mushrooms
	07095930	Fresh or frozen enoki mushroom
	07095940	Fresh or frozen straw mushrooms
	07095950	Fresh or frozen portobello mushrooms
	07095960	Fresh or frozen truffle mushrooms

07095990	Other fresh or frozen mushrooms
07096000	Fresh or frozen peppers, including bell peppers
07097000	Fresh or frozen spinach
07099300	Fresh or frozen olive oil
07099300	Fresh or frozen pumpkin, gourds
07099910	Fresh or frozen bamboo shoots
07099990	Fresh or frozen vegetables not listed
07101000	Frozen potatoes
07102100	Frozen peas
07102210	Frozen red beans
07102290	Frozen cowpeas and other vegetable beans
07102900	Other frozen legume vegetables
07103000	Frozen spinach
07104000	Frozen sweet corn
07108010	Frozen matsutake mushroom
07108020	Frozen garlic & garlic scapes (green garlic)
07108030	Frozen garlic
07108040	Frozen porcini mushrooms
07108090	Frozen unlisted vegetables
07109000	Frozen assorted vegetables
07112000	Temporarily preserved oil olives
07114000	Temporarily preserved cucumbers and gherkins
07115112	Brined baby white mushrooms
07115119	Other mushrooms of the genus Paramecium in brine
07115190	Other mushrooms of the genus Paramecium temporarily preserved
07115911	Matsutake mushrooms in brine
07115919	Other mushrooms and truffles in brine
07115990	Other mushrooms and truffles temporarily preserved
07119031	Bamboo shoots, brine
07119034	Garlic in brine
07119039	Other vegetables and assorted vegetables in brine
07119090	Vegetables and assorted vegetables not listed for temporary preservation
07122000	Dried onions
07123100	Dried mushrooms of the genus Paramecium
07123200	Dried wood fungus
07123300	Dried silver fungus
07123910	Dried shiitake mushrooms
07123920	Dried enoki mushrooms
07123930	Dried straw mushrooms
07123940	Dried portobello mushrooms
07123950	Dried porcini mushrooms
07123990	Dried mushrooms and truffles not listed
07129010	Dried shredded bamboo shoots

	07129020	Dried vetches (dried vetch)
	07129030	Golden needle (cauliflower)
	07129040	Dried fiddleheads
	07129050	Dried garlic
	07129060	Dried bell pepper
	07129091	Horseradish
	07129099	Not listed dried vegetables and assorted vegetables
Bean	07131010	Seed peas
	07131090	Other dry peas
	07132010	Chickpeas for seed
	07132090	Other dried chickpeas
	07133190	Other dried mung beans
	07133210	Red beans (adzuki beans) for seed, podless, dried
	07133290	Other red lentils (adzuki beans), off-pod, dry
	07133310	Kidney beans for seed
	07133390	Other dried kidney beans
	07133500	Cowpeas (cowpeas), dried
	07133900	Dry beans of unlisted cowpea genus and navy bean genus
	07134010	Lentils for seed use
	07134090	Other dry lentils
	07135010	Broad beans for seed
	07135090	Other dried fava beans
	07136090	Other dried wood beans (genus Mucuna)
	07139010	Other dry podded beans for seed
	07139090	Other dry podded beans, except for seed
Tuber	07061000	Fresh or frozen carrots and radishes
	07069000	Other fresh or frozen edible roots and tubers
	07141010	Fresh sweet potatoes
	07141020	Dried cassava
	07142019	Other fresh sweet potatoes
	07142020	Dried sweet potatoes
	07142030	Chilled or frozen sweet potatoes
	07143000	Yam
	07144000	Taro (Taro spp.)
	07149010	Water chestnut
	07149021	Roots for seed
	07149029	Other roots
	07149090	Fresh, cold, frozen or dried bamboo taro, orchid tubers, chrysanthemum taro and unlisted roots containing high starch or inulin; West Valley stem pith
Fruit	08011100	Dried coconut
	08011200	Coconut without inner shell removed
	08011990	Other coconut
	08012100	Brazil nuts unshelled

08012200	Brazil nuts in shell
08013100	Unshelled cashew nuts
08013200	Cashew nuts in shell
08021100	Unhusked apricots
08021200	Apricot kernels
08022100	Hazelnuts unshelled
08022200	Hazelnut kernels
08023100	Unshelled walnuts
08023200	Walnut kernels
08024110	Unshelled chestnuts
08024190	Other unshelled chestnuts
08024210	Chestnuts in shell
08024290	Other chestnuts in shell
08025100	Unshelled pistachios (pistachios)
08025200	Pistachios in shell (pistachios)
08026190	Other unshelled macadamia nuts (macadamia)
08026200	Macadamia nuts in shell (macadamia nuts)
08028000	Betel nuts
08029020	White nuts
08029030	Pine nuts
08029090	Fresh or dried nuts not listed
08031000	Fresh or dried plantain
08039000	Other fresh or dried bananas, except plantains
08041000	Fresh or dried dates
08042000	Fresh or dried figs
08043000	Fresh or dried pineapple
08044000	Fresh or dried avocados
08045010	Guava, fresh or dried
08045020	Fresh or dried mangoes
08045030	Fresh or dried mangosteen
08051000	Fresh or dried oranges
08052010	Fresh or dried banana mandarins
08052020	Broad-leaved mandarin oranges
08052090	Other fresh or dried citrus and hybrid citrus
08054000	Grapefruit, including pomelo
08055000	Lemons and limes
08059000	Citrus fruits not listed
08061000	Fresh grapes
08062000	Raisins
08071100	Fresh watermelons
08071910	Fresh cantaloupes
08071990	Other fresh melons
08072000	Fresh papayas

	08081000	Fresh apples
	08083010	Fresh duck pears, snow pears
	08083020	Fresh balsam pears
	08083090	Other fresh pears
	08091000	Fresh apricots
	08092900	Other fresh cherries
	08093000	Fresh peaches, including nectarines
	08094000	Fresh plums and plums
	08101000	Fresh strawberries
	08102000	Fresh raspberries, blackberries, mulberries and loganberries
	08104000	Fresh cranberries and lingonberries
	08105000	Fresh kiwifruit
	08106000	Fresh durian
	08107000	Fresh persimmons
	08109010	Fresh lychees
	08109030	Fresh longans
	08109040	Fresh rambutans
	08109050	Fresh lychees
	08109060	Fresh poppy peach
	08109070	Fresh lotus mist
	08109080	Fresh dragon fruit
	08109090	Fresh fruit not listed
	08111000	Frozen strawberries
	08112000	Frozen raspberries, blackberries, blackberries, mulberries, loganberries, currants and galangal
	08119010	Frozen chestnuts, unhulled
	08119090	Other unlisted frozen fruits and nuts
	08121000	Temporarily preserved cherries
	08129000	Other temporarily preserved fruits and nuts
	08131000	Dried apricots
	08132000	Dried plums and prunes
	08133000	Dried apples
	08134010	Dried longan, meat
	08134020	Persimmon cakes
	08134030	Red dates
	08134040	Dried lychees
	08134090	Dried fruits not listed
	08135000	Assorted nuts or dried fruits of this chapter
	08140000	Citrus fruits or melons (including watermelon) rind, fresh, frozen, dried or temporarily preserved with brine, sulfite water or other preservatives
	10011100	Durum wheat for seed
Wheat	10011900	Other durum wheat
	10019100	Other wheat and mixed wheat for seed

	10019900	Other wheat and mixed wheat, except for seed
	10021000	Rye for seed
	10031000	Barley for seed
	10039000	Other barley
	10041000	Oats for seed
	10049000	Other oats
	10086090	Other rye
	11010000	Wheat and mixed wheat fine flour
	11031100	Wheat coarse grain, coarse flour
	11031910	Oats coarse grain, coarse flour
	11041200	Oats, hydraulic or produced
	11041910	Barley, hydraulic or produced
	11042200	Oats otherwise processed
	11042910	Barley otherwise processed
	11081100	Wheat starch
Corn	10051000	Corn for seed
	10059000	Maize, except for seed
	11022000	Corn fine meal
	11031300	Corn coarse grain, coarse meal
	11042300	Corn otherwise processed
	11081200	Corn starch
Rice	10061011	Indica rice for seed
	10061019	Other seed rice
	10061091	Indica rice
	10061099	Other rice
	10062010	Indica brown rice
	10062090	Other brown rice
	10063010	Indica fine rice
	10063090	Other fine rice
	10064010	Indica rice broken rice
	10064090	Other broken rice
	10071000	Edible sorghum for seed
	10079000	Other edible sorghum
	10081000	Buckwheat
	10082900	Other grains
	10089010	Other seed grains
	10089090	Unlisted grains
	11029011	Indica rice fine flour
	11029019	Other rice fine flour
	11029090	Fine flour of unlisted grains
	11031921	Indica rice coarse grains, coarse flour
	11031929	Other rice coarse grains, coarse flour
	11031990	Other cereals coarse grains, coarse flour

	11032090	Other cereal grains
	11041990	Other rolled or produced grains
	11042990	Other processed unlisted grains
Oil crops	12011000	Soybeans for seed
	12019010	Yellow soybeans, except for seed
	12019020	Black soybeans, except for seed
	12019030	Green soybeans, except for seed
	12019090	Other soybeans, except for seed
	12023000	Peanuts for seed
	12024100	Peanuts, other than seed, not shelled
	12024200	Peanuts, other shelled, whether broken or unbroken
	12040000	Flaxseed, broken or unbroken
	12051010	Low erucic acid rapeseed for seed
	12051090	Other low erucic acid rapeseed
	12059010	Rapeseed, other than seed
	12059090	Canola seeds, not listed
	12060010	Sunflower seeds for seed
	12060090	Other sunflower seeds
	12072100	Cottonseed for seed
	12072900	Other cotton seeds
	12073010	Castor seed for seed
	12073090	Other castor seeds
	12074010	Sesame seeds for seeds
	12074090	Other sesame seeds
	12075010	Mustard seeds for seeds
	12075090	Other mustard seeds
	12076010	Safflower seeds for seeds
	12076090	Other safflower seeds
	12077010	Sweet melon seeds for seeds
	12077091	Black melon seeds
	12077092	Red melon seeds
	12077099	Melon seeds not listed, except for seeds
	12079100	Poppy seeds
	12079910	Other seeds with oil seeds and fruits
	12079999	Oil-bearing kernels and fruits not listed
	12081000	Soybean flour
	12089000	Other oil-bearing kernels and fruits fine and coarse powder, except mustard powder

Source: [2].

Table S3. Embodied GHG emissions in the exports of China's agricultural products during 1995-2015 (Unit: 10^4 t CO_2-eq).

Year	Corn	Rice	Wheat	Sugar	Tubers	Oil crops	Beans	Fruits	Vegetables	Beef	Pork	Mutton	Poultry	Milk	Eggs
1995	5.35	49.39	37.77	0.67	5.73	128.54	39.12	301.78	55.94	31.53	125.23	11.16	52.74	7.37	12.07
1996	10.52	63.32	96.83	0.58	5.87	119.60	24.06	318.87	59.86	20.43	113.80	2.44	59.03	7.23	17.73
1997	390.69	273.81	71.04	0.35	5.61	67.17	26.50	464.67	64.23	57.75	100.99	1.22	55.73	8.53	24.58
1998	293.15	994.74	30.51	0.57	6.66	69.23	21.04	352.32	79.91	73.64	96.85	1.24	41.76	8.52	22.41
1999	84.49	622.86	11.75	0.56	7.39	101.03	32.35	332.34	94.15	24.12	73.51	1.10	53.26	11.06	21.00
2000	646.94	639.24	44.43	0.71	7.67	110.60	26.13	328.29	96.58	16.10	76.46	2.45	63.16	11.82	26.75
2001	374.48	386.79	81.98	0.72	8.11	120.39	30.95	420.20	118.91	16.60	94.80	1.64	63.17	9.64	25.48
2002	753.68	403.58	83.51	0.60	12.51	125.56	36.91	508.02	154.34	13.90	111.82	4.38	58.31	11.60	31.94
2003	1060.26	546.99	137.39	0.53	11.73	131.82	44.35	501.13	137.62	9.29	132.08	20.13	51.41	10.05	35.79
2004	202.27	227.61	76.70	0.51	13.39	121.85	38.37	590.83	144.81	15.47	168.11	51.11	25.22	12.67	33.17
2005	671.89	240.82	25.35	0.57	14.58	136.56	43.99	625.65	152.48	16.97	142.51	56.49	35.02	16.42	33.16
2006	249.42	308.37	76.99	0.51	14.51	111.44	38.59	732.43	166.00	23.76	153.35	65.12	28.67	16.50	31.83
2007	405.34	358.00	178.58	0.52	14.08	134.74	37.43	810.34	191.83	50.62	93.01	36.10	29.61	44.30	29.01
2008	21.65	255.14	5.94	0.76	14.73	146.62	46.72	552.35	205.78	23.66	73.59	25.50	30.57	38.20	34.96
2009	12.23	217.14	6.41	0.65	14.60	110.58	47.71	639.29	199.65	12.70	71.91	12.11	30.55	10.98	32.08
2010	11.20	151.34	7.77	0.91	16.13	71.92	46.74	664.47	198.04	25.80	81.76	19.91	35.01	6.88	35.51
2011	7.68	164.38	8.19	0.88	17.91	87.49	44.05	641.81	236.51	26.60	68.98	7.90	34.72	18.04	35.28
2012	4.65	98.52	4.08	0.99	21.13	99.72	48.54	571.26	229.50	18.25	67.45	3.20	32.59	15.06	35.15
2013	3.30	135.87	2.53	1.09	19.27	73.21	38.11	595.23	232.11	12.56	72.81	0.81	33.56	7.84	32.33
2014	1.43	119.66	1.34	1.07	20.18	83.64	25.74	537.89	251.09	12.40	80.05	2.28	36.50	11.16	32.23
2015	1.79	86.09	0.58	1.24	20.43	65.58	21.15	529.12	254.54	10.29	72.28	2.34	38.42	7.67	33.24

Source: [2].

Table S4. The proportion of embodied GHG emissions in the exports during 1995-2015. **.**

Year	Corn	Rice	Wheat	Sugar	Oil crops	Beans	Tubers	Fruits	Vegetables	Beef	Pork	Mutton	Poultry	Milk	Eggs
1995	0.6%	5.7%	4.4%	0.1%	14.9%	4.5%	0.7%	34.9%	6.5%	3.6%	14.5%	1.3%	6.1%	0.9%	1.4%
1996	1.1%	6.9%	10.5%	0.1%	13.0%	2.6%	0.6%	34.7%	6.5%	2.2%	12.4%	0.3%	6.4%	0.8%	1.9%
1997	24.2%	17.0%	4.4%	0.0%	4.2%	1.6%	0.3%	28.8%	4.0%	3.6%	6.3%	0.1%	3.5%	0.5%	1.5%
1998	14.0%	47.5%	1.5%	0.0%	3.3%	1.0%	0.3%	16.8%	3.8%	3.5%	4.6%	0.1%	2.0%	0.4%	1.1%
1999	5.7%	42.3%	0.8%	0.0%	6.9%	2.2%	0.5%	22.6%	6.4%	1.6%	5.0%	0.1%	3.6%	0.8%	1.4%
2000	30.8%	30.5%	2.1%	0.0%	5.3%	1.2%	0.4%	15.7%	4.6%	0.8%	3.6%	0.1%	3.0%	0.6%	1.3%
2001	21.4%	22.1%	4.7%	0.0%	6.9%	1.8%	0.5%	24.0%	6.8%	0.9%	5.4%	0.1%	3.6%	0.5%	1.5%
2002	32.6%	17.5%	3.6%	0.0%	5.4%	1.6%	0.5%	22.0%	6.7%	0.6%	4.8%	0.2%	2.5%	0.5%	1.4%
2003	37.5%	19.3%	4.9%	0.0%	4.7%	1.6%	0.4%	17.7%	4.9%	0.3%	4.7%	0.7%	1.8%	0.4%	1.3%
2004	11.7%	13.2%	4.5%	0.0%	7.1%	2.2%	0.8%	34.3%	8.4%	0.9%	9.8%	3.0%	1.5%	0.7%	1.9%
2005	30.4%	10.9%	1.1%	0.0%	6.2%	2.0%	0.7%	28.3%	6.9%	0.8%	6.4%	2.6%	1.6%	0.7%	1.5%
2006	12.4%	15.3%	3.8%	0.0%	5.5%	1.9%	0.7%	36.3%	8.2%	1.2%	7.6%	3.2%	1.4%	0.8%	1.6%
2007	16.8%	14.8%	7.4%	0.0%	5.6%	1.6%	0.6%	33.6%	7.9%	2.1%	3.9%	1.5%	1.2%	1.8%	1.2%
2008	1.5%	17.3%	0.4%	0.1%	9.9%	3.2%	1.0%	37.4%	13.9%	1.6%	5.0%	1.7%	2.1%	2.6%	2.4%
2009	0.9%	15.3%	0.5%	0.0%	7.8%	3.4%	1.0%	45.1%	14.1%	0.9%	5.1%	0.9%	2.2%	0.8%	2.3%
2010	0.8%	11.0%	0.6%	0.1%	5.2%	3.4%	1.2%	48.4%	14.4%	1.9%	6.0%	1.4%	2.5%	0.5%	2.6%
2011	0.5%	11.7%	0.6%	0.1%	6.2%	3.1%	1.3%	45.8%	16.9%	1.9%	4.9%	0.6%	2.5%	1.3%	2.5%
2012	0.4%	7.9%	0.3%	0.1%	8.0%	3.9%	1.7%	45.7%	18.4%	1.5%	5.4%	0.3%	2.6%	1.2%	2.8%
2013	0.3%	10.8%	0.2%	0.1%	5.8%	3.0%	1.5%	47.2%	18.4%	1.0%	5.8%	0.1%	2.7%	0.6%	2.6%
2014	0.1%	9.8%	0.1%	0.1%	6.9%	2.1%	1.7%	44.2%	20.6%	1.0%	6.6%	0.2%	3.0%	0.9%	2.6%
2015	0.2%	7.5%	0.1%	0.1%	5.7%	1.8%	1.8%	46.2%	22.2%	0.9%	6.3%	0.2%	3.4%	0.7%	2.9%

Source: [2].

Table S5. The embodied GHG emissions in the exports of China's agricultural products to major countries in 2015 (Unit: 10^4 t CO_2-eq).

Country (region)	Export	Proportion
Mongolia	281.26	24.57%
Hong Kong	175.68	15.35%
Singapore	151.86	13.27%
South Korea	104.09	9.09%
Japan	74.04	6.47%
Vietnam	36.46	3.19%
Netherlands	30.51	2.66%
Germany	29.65	2.59%
Thailand	27.61	2.41%
Russian	23.25	2.03%
Others	210.34	18.37%

Source: [2].

Table S6. The embodied GHG emissions in the imports of China's agricultural products during 1995-2015 (Unit: 10^4 t CO_2-eq).

Year	Corn	Rice	Wheat	Sugar	Tubers	Oil crops	Beans	Fruits	Vegetables	Beef	Pork	Mutton	Poultry	Milk	Eggs
1995	59.19	383.24	502.20	0.00	3.87	15.81	0.15	10.98	0.19	10.41	10.35	4.56	40.80	12.52	0.22
1996	5.64	189.76	556.91	0.00	0.80	47.50	4.25	17.97	0.41	11.76	2.43	9.11	48.13	14.58	0.41
1997	0.14	62.72	238.14	0.00	2.28	128.84	2.09	24.25	0.53	9.70	4.61	11.28	30.94	18.67	0.87
1998	5.85	68.24	190.35	0.00	2.28	183.98	1.71	23.21	0.83	18.04	18.08	25.54	27.71	21.84	0.26
1999	1.34	48.86	214.11	0.00	3.05	302.62	2.06	44.19	1.76	29.10	88.34	29.01	116.55	25.28	0.13
2000	0.55	24.88	289.67	0.04	25.69	1340.48	10.58	98.40	4.84	53.44	154.30	48.90	129.10	21.88	0.03
2001	1.22	77.19	205.55	0.00	12.99	713.20	2.51	99.48	3.02	41.61	139.97	68.95	107.80	28.31	0.09
2002	0.33	62.58	203.76	0.00	11.91	566.71	2.09	122.02	2.00	52.89	160.02	94.07	86.98	39.20	0.09
2003	0.08	68.28	90.41	0.00	17.09	986.64	1.28	92.96	1.87	74.77	224.15	93.02	97.12	47.38	0.06
2004	0.14	197.69	537.78	0.00	23.51	957.10	1.79	154.66	2.04	100.96	209.38	88.49	23.10	52.67	0.00
2005	0.48	134.19	349.03	0.00	22.60	1291.66	3.98	68.75	2.98	31.92	137.87	109.72	51.43	47.99	0.01
2006	1.05	189.22	241.71	0.00	34.05	1447.67	5.44	180.20	7.87	12.47	137.41	96.89	81.40	53.21	0.09
2007	0.63	126.22	71.05	0.00	33.46	1535.27	4.73	105.49	0.58	21.58	296.03	122.97	108.23	45.46	0.02
2008	0.68	88.54	96.21	0.01	14.77	1850.48	4.89	91.64	0.58	16.39	605.17	145.88	111.84	50.89	0.22
2009	3.39	143.32	23.61	0.01	153.93	1443.01	5.85	106.65	0.65	47.82	355.14	177.62	106.24	91.49	0.06
2010	17.99	111.23	278.15	0.01	39.22	2760.83	9.03	117.77	1.67	88.75	612.60	153.46	61.15	118.07	0.16
2011	19.58	131.62	248.61	0.01	36.58	2685.52	11.11	158.32	1.25	84.88	943.81	217.22	49.12	142.40	0.08
2012	57.50	462.10	554.22	0.01	51.73	3066.12	10.32	174.30	2.41	159.62	888.76	348.76	65.82	182.60	0.00
2013	36.41	629.86	467.85	0.01	50.49	3384.75	15.04	156.00	2.10	489.72	856.81	675.55	78.95	251.82	0.03
2014	29.88	1605.84	678.00	0.02	57.94	3781.09	12.06	175.11	1.88	572.88	850.05	742.06	60.34	285.61	0.00
2015	45.53	2681.67	827.65	0.02	63.51	4327.18	13.76	266.25	2.40	738.26	924.88	591.14	46.86	243.78	0.00

Source: [2].

Table S7. The proportion of embodied GHG emissions in the imports during 1995-2015.

Year	Corn	Rice	Wheat	Sugar	Oil crops	Beans	Tubers	Fruits	Vegetables	Beef	Pork	Mutton	Poultry	Milk	Eggs
1995	5.6%	36.3%	47.6%	0.0%	1.5%	0.0%	0.4%	1.0%	0.0%	1.0%	1.0%	0.4%	3.9%	1.2%	0.0%
1996	0.6%	20.9%	61.2%	0.0%	5.2%	0.5%	0.1%	2.0%	0.0%	1.3%	0.3%	1.0%	5.3%	1.6%	0.0%
1997	0.0%	11.7%	44.5%	0.0%	24.1%	0.4%	0.4%	4.5%	0.1%	1.8%	0.9%	2.1%	5.8%	3.5%	0.2%
1998	1.0%	11.6%	32.4%	0.0%	31.3%	0.3%	0.4%	3.9%	0.1%	3.1%	3.1%	4.3%	4.7%	3.7%	0.0%
1999	0.1%	5.4%	23.6%	0.0%	33.4%	0.2%	0.3%	4.9%	0.2%	3.2%	9.7%	3.2%	12.9%	2.8%	0.0%
2000	0.0%	1.1%	13.2%	0.0%	60.9%	0.5%	1.2%	4.5%	0.2%	2.4%	7.0%	2.2%	5.9%	1.0%	0.0%
2001	0.1%	5.1%	13.7%	0.0%	47.5%	0.2%	0.9%	6.6%	0.2%	2.8%	9.3%	4.6%	7.2%	1.9%	0.0%
2002	0.0%	4.5%	14.5%	0.0%	40.3%	0.1%	0.8%	8.7%	0.1%	3.8%	11.4%	6.7%	6.2%	2.8%	0.0%
2003	0.0%	3.8%	5.0%	0.0%	55.0%	0.1%	1.0%	5.2%	0.1%	4.2%	12.5%	5.2%	5.4%	2.6%	0.0%
2004	0.0%	8.4%	22.9%	0.0%	40.7%	0.1%	1.0%	6.6%	0.1%	4.3%	8.9%	3.8%	1.0%	2.2%	0.0%
2005	0.0%	6.0%	15.5%	0.0%	57.3%	0.2%	1.0%	3.1%	0.1%	1.4%	6.1%	4.9%	2.3%	2.1%	0.0%
2006	0.0%	7.6%	9.7%	0.0%	58.2%	0.2%	1.4%	7.2%	0.3%	0.5%	5.5%	3.9%	3.3%	2.1%	0.0%
2007	0.0%	5.1%	2.9%	0.0%	62.1%	0.2%	1.4%	4.3%	0.0%	0.9%	12.0%	5.0%	4.4%	1.8%	0.0%
2008	0.0%	2.9%	3.1%	0.0%	60.1%	0.2%	0.5%	3.0%	0.0%	0.5%	19.7%	4.7%	3.6%	1.7%	0.0%
2009	0.1%	5.4%	0.9%	0.0%	54.3%	0.2%	5.8%	4.0%	0.0%	1.8%	13.4%	6.7%	4.0%	3.4%	0.0%
2010	0.4%	2.5%	6.4%	0.0%	63.2%	0.2%	0.9%	2.7%	0.0%	2.0%	14.0%	3.5%	1.4%	2.7%	0.0%
2011	0.4%	2.8%	5.3%	0.0%	56.8%	0.2%	0.8%	3.3%	0.0%	1.8%	20.0%	4.6%	1.0%	3.0%	0.0%
2012	1.0%	7.7%	9.2%	0.0%	50.9%	0.2%	0.9%	2.9%	0.0%	2.6%	14.8%	5.8%	1.1%	3.0%	0.0%
2013	0.5%	8.9%	6.6%	0.0%	47.7%	0.2%	0.7%	2.2%	0.0%	6.9%	12.1%	9.5%	1.1%	3.5%	0.0%
2014	0.3%	18.1%	7.7%	0.0%	42.7%	0.1%	0.7%	2.0%	0.0%	6.5%	9.6%	8.4%	0.7%	3.2%	0.0%
2015	0.4%	24.9%	7.7%	0.0%	40.2%	0.1%	0.6%	2.5%	0.0%	6.9%	8.6%	5.5%	0.4%	2.3%	0.0%

Source: [2].

Table S8. The embodied GHG emissions in the imports of China's agricultural products from major countries in 2015 (Unit: 10^4 t CO_2-eq).

Country (region)	Import	Proportion
U.S.	3117.61	28.94%
Brazil	2111.45	19.60%
Australia	1413.09	13.12%
New Zealand	727.02	6.75%
Argentina	567.51	5.27%
Uruguay	516.59	4.79%
Vietnam	351.00	3.26%
Thailand	324.17	3.01%
Canada	311.14	2.89%
Germany	205.10	1.90%
Others	1128.21	10.47%

Source: [2].

Table S9. The variation trend of net import and export volume of China's agricultural products (Unit: 10^4 t).

Year	Export	Import	Net	Total
1995	-553.77	2190.51	1636.74	2744.28
1996	-558.78	1331.47	772.70	1890.25
1997	-1253.29	870.44	-382.85	2123.74
1998	-1321.90	1007.15	-314.75	2329.04
1999	-1256.25	1265.31	9.07	2521.56
2000	-1922.48	1931.71	9.23	3854.19
2001	-1496.75	2343.97	847.21	3840.72
2002	-2216.07	1888.72	-327.35	4104.79
2003	-3053.58	2800.70	-252.88	5854.27
2004	-1352.59	3616.72	2264.13	4969.32
2005	-1992.87	3912.89	1920.02	5905.75
2006	-1598.60	4076.26	2477.66	5674.86
2007	-2096.41	4150.07	2053.66	6246.48
2008	-1360.28	4686.19	3325.90	6046.47
2009	-1319.57	6043.16	4723.59	7362.72
2010	-1275.45	7422.07	6146.62	8697.51
2011	-1350.13	7205.73	5855.60	8555.86
2012	-1303.91	9074.94	7771.03	10378.85
2013	-1305.93	9827.65	8521.73	11133.58
2014	-1262.74	11480.72	10217.98	12743.46
2015	-1262.74	13943.71	12680.98	15206.45

Source: [2].

Table S10. The variation trend of embodied GHG emissions in the net import and export of China's agricultural products (Unit: 10^4 t CO_2-eq).

Year	Export	Import	Net	Total
1995	-864.39	1054.49	190.11	1918.88
1996	-920.16	909.68	-10.48	1829.84
1997	-1612.87	535.05	-1077.82	2147.93
1998	-2092.53	587.91	-1504.62	2680.45
1999	-1470.98	906.40	-564.58	2377.38
2000	-2097.33	2202.79	105.46	4300.12
2001	-1753.86	1501.89	-251.97	3255.75
2002	-2310.65	1404.66	-906.00	3715.31
2003	-2830.56	1795.12	-1035.44	4625.68
2004	-1722.08	2349.33	627.25	4071.41
2005	-2212.45	2252.61	40.16	4465.06
2006	-2017.49	2488.68	471.18	4506.17
2007	-2413.51	2471.72	58.21	4885.23
2008	-1476.18	3078.20	1602.03	4554.38
2009	-1418.59	2658.80	1240.20	4077.39
2010	-1373.38	4370.08	2996.70	5743.46
2011	-1400.42	4730.10	3329.68	6130.52
2012	-1250.08	6024.27	4774.19	7274.34
2013	-1260.63	7095.39	5834.76	8356.02
2014	-1216.67	8852.77	7636.10	10069.44
2015	-1144.76	10772.88	9628.13	11917.64

Source: [2].

Table S11. The embodied GHG emissions in the net imports of China's agricultural products with major countries from 1995 to 2015 (Unit: 10^4 t CO_2-eq).

Country (region)	Corn	Rice	Wheat	Sugar	Oil crops	Beans	Tubers	Fruits	Vegetables	Beef	Pork	Mutton	Poultry	Milk	Eggs
South Korea	-3772.35	-783.09	-445.18	-0.01	-848.98	-77.72	81.20	-39.08	-205.39	-0.75	-4.76	0.03	-8.44	1.23	-1.50
Mongolia	0.00	-0.49	-420.15	-0.03	169.85	-0.03	-8.91	-4184.87	-335.11	6.88	-7.73	0.22	-0.65	-6.69	-0.04
Singapore	0.01	-0.01	0.07	-0.09	-40.38	0.00	-47.21	-3346.13	-530.26	0.00	-66.67	0.00	-6.69	0.01	-7.16
Japan	-621.45	-811.10	-0.60	-9.11	-621.69	-95.13	-68.00	-140.62	-476.50	-1.42	-0.30	-0.49	-183.60	-0.18	-112.18
Hong Kong	0.01	-3.13	0.04	-0.04	0.02	0.01	-31.25	-403.24	-446.60	-124.30	-1493.76	0.00	-436.04	-111.70	-324.41
New Zealand	-3.21	-0.01	9.85	0.00	-21.57	1.73	-0.25	460.23	-2.67	572.99	0.07	2229.02	0.51	913.25	-0.35
Argentina	6.36	15.59	27.49	0.00	5273.57	-0.19	0.00	0.99	-0.76	105.99	0.04	0.00	62.34	16.42	0.00
Australia	-1.15	399.08	4805.43	-0.22	418.95	4.42	-0.04	3.48	-11.06	1045.10	4.00	1230.24	5.74	106.28	-5.23
Brazil	0.27	-1.66	0.00	0.00	11724.70	-28.20	0.00	-0.43	-33.45	110.20	3.24	0.00	259.38	0.24	0.00
U.S.	203.83	2684.35	705.77	-0.81	10597.32	0.13	-1.06	47.44	-49.97	139.12	2638.83	0.22	968.59	262.55	-67.31

Source: [2].

Table S12. The embodied GHG emissions in the net imports of China's agricultural products with major countries in 2015 (Unit: 10^2 t CO_2-eq).

Country (region)	Corn	Rice	Wheat	Sugar	Oil crops	Beans	Tubers	Fruits	Vegetables	Beef	Pork	Mutton	Poultry	Milk	Eggs
South Korea	0.26	-4784.49	18.87	0.00	-2642.50	-222.39	-473.50	-133.13	-2061.39	0.00	0.00	0.00	0.00	45.46	-8.74
Mongolia	0.00	0.00	89.58	0.00	7232.90	-0.86	-59.21	-21056.74	-3034.34	0.00	-90.73	-0.19	-20.67	-13.43	-0.43
Singapore	0.00	0.00	0.00	0.00	-123.97	0.00	-348.51	-10762.62	-3847.08	0.00	-65.39	0.00	0.00	0.00	-38.68
Japan	0.22	-2415.18	4.48	-26.12	-884.57	-325.51	-321.15	-417.62	-2438.30	0.00	0.00	0.00	-0.79	0.01	-544.93
Hong Kong	0.00	0.00	0.00	0.00	0.00	0.00	-203.94	-2344.36	-3371.17	-367.14	-6598.64	0.00	-2333.56	-419.86	-1929.64
New Zealand	-4.13	0.00	-1.90	0.00	-65.58	9.57	-1.60	6590.56	-106.02	15202.41	0.00	38570.77	0.00	11828.21	-3.78
Argentina	0.02	1560.64	161.73	-0.02	48227.76	-0.72	0.00	1.15	-2.42	6228.25	0.00	0.00	300.26	270.56	0.00
Australia	-5.68	30864.43	62327.19	-3.05	3586.31	48.20	-0.24	335.19	-56.84	22610.33	0.00	19940.92	0.00	1435.72	-48.41
Brazil	-0.11	-12.60	0.00	0.04	200383.46	-51.58	0.00	-7.38	-230.47	7275.93	96.18	0.00	3388.29	0.00	0.00
U.S.	509.87	167572.00	2342.02	-0.12	119268.34	84.93	-6.20	316.19	-258.59	0.16	17632.31	0.00	508.45	3087.15	-290.03

Source: [2].

Table S13. The export volume of China's agricultural products from 1995 to 2015 (Unit: 10^4 t).

Year	Corn	Rice	Wheat	Sugar	Tuber	Oil crops	Bean	Fruits	Vegetables	Beef	Pork	Mutton	Poultry	Milk	Egg
1995	13.82	26.23	23.31	8.72	18.32	96.82	104.12	52.15	117.47	5.07	41.77	0.83	38.90	3.05	3.19
1996	25.29	39.83	57.25	8.36	15.79	73.58	54.71	59.46	131.87	3.56	37.91	0.24	43.31	3.14	4.48
1997	664.80	117.03	46.88	4.86	12.67	44.95	59.36	76.63	133.73	5.01	34.03	0.17	42.88	3.90	6.40
1998	469.77	387.08	28.73	7.92	18.99	47.35	47.25	71.99	159.13	6.36	33.03	0.32	34.30	3.75	5.95
1999	434.12	283.92	17.45	8.65	20.46	71.27	81.08	80.55	177.57	3.60	25.66	0.38	42.76	4.04	4.73
2000	1054.23	307.92	19.41	10.37	22.62	80.40	57.20	88.61	190.09	3.38	26.34	0.46	49.94	4.80	6.73
2001	606.20	199.32	71.93	10.74	24.69	93.93	71.89	89.20	237.17	3.64	30.59	0.32	46.80	4.27	6.07
2002	1177.11	210.98	98.54	7.72	34.95	101.86	86.04	123.33	281.87	2.73	35.62	0.52	41.06	5.10	8.66
2003	1650.96	295.33	253.33	8.50	42.32	103.54	104.11	162.93	338.51	2.23	40.83	1.27	35.04	4.89	9.79
2004	242.91	119.47	114.48	8.22	52.24	93.12	79.54	191.91	364.21	3.10	49.75	2.88	15.68	6.01	9.08
2005	878.14	80.70	62.31	8.95	63.56	110.56	86.79	219.48	395.31	3.31	43.35	3.23	21.53	6.98	8.66
2006	328.44	136.34	153.15	8.27	68.16	93.61	80.14	215.30	429.68	2.78	44.80	3.60	18.34	7.49	8.50
2007	526.31	169.22	321.96	6.63	63.88	100.50	85.95	262.42	482.05	4.16	30.04	2.26	19.20	13.46	8.37
2008	71.86	117.41	34.68	8.69	62.35	95.35	103.12	306.49	488.21	3.18	25.23	1.51	19.87	12.06	10.28
2009	41.94	92.76	27.29	7.36	63.25	81.60	109.56	349.65	485.66	1.39	24.99	1.02	19.94	3.68	9.48
2010	49.39	74.23	30.35	10.37	69.37	60.12	100.00	322.28	489.10	4.02	27.95	1.41	22.98	3.38	10.51
2011	36.23	74.17	34.69	10.28	71.99	64.44	99.19	312.26	577.91	4.03	23.77	0.86	23.08	6.79	10.44
2012	36.29	46.80	29.59	11.29	81.72	77.51	98.44	328.23	530.70	2.89	23.51	0.54	21.66	4.49	10.25
2013	17.46	60.78	28.59	12.77	80.52	65.65	84.18	318.76	574.25	1.83	25.45	0.35	22.41	3.61	9.33
2014	7.65	55.80	19.64	13.29	81.97	65.11	54.67	289.05	607.85	1.90	27.41	0.46	24.50	3.99	9.46
2015	8.49	39.78	12.57	14.87	86.74	63.33	49.93	304.29	616.30	1.69	25.17	0.39	26.08	3.33	9.76

Source: [2].

Table S14. The major export trading partners in 2015 (Unit: 10^4 t).

Country (region)	Export volume	Proportion
Hong Kong	167.05	13.23%
Vietnam	128.21	10.15%
Japan	118.03	9.35%
South Korea	100.22	7.94%
Malaysia	94.15	7.46%
Russian	82.85	6.56%
Indonesia	81.15	6.43%
Thailand	80.14	6.35%
U.S.	37.74	2.99%
Others	393.22	29.56%

Source: [2].

Table S15. The import volume of China's agricultural products from 1995 to 2015 (Unit: 10^4 t).

Year	Corn	Rice	Wheat	Sugar	Tuber	Oil crops	Bean	Fruits	Vegetables	Beef	Pork	Mutton	Poultry	Milk	Egg
1995	526.55	164.53	1349.48	0.02	45.78	41.72	1.00	23.72	1.03	0.90	1.72	0.17	26.41	7.22	0.25
1996	44.91	77.71	961.55	0.01	8.06	112.21	19.31	64.54	2.14	1.03	0.36	0.33	31.51	7.75	0.05
1997	1.52	35.96	380.70	0.02	27.88	297.02	12.53	76.47	3.86	0.79	0.66	0.41	21.18	11.36	0.08
1998	25.37	26.04	337.32	0.05	30.17	461.47	10.19	75.89	4.85	1.52	2.67	0.92	19.55	11.08	0.05
1999	8.22	19.21	312.89	0.09	37.30	694.14	5.68	68.66	5.68	2.46	13.48	1.05	80.08	16.34	0.03
2000	0.55	24.88	289.67	0.04	25.69	1340.48	10.58	98.40	4.84	3.85	23.81	1.79	85.22	21.88	0.03
2001	4.26	29.45	311.08	0.02	195.06	1570.76	17.07	96.35	2.95	3.69	20.43	2.54	70.71	19.56	0.02
2002	0.98	24.14	260.36	0.08	176.11	1195.37	12.62	101.69	2.61	5.27	21.96	3.50	57.63	26.38	0.02
2003	0.18	26.04	182.77	0.06	236.84	2099.53	6.92	107.94	2.65	6.97	31.21	3.44	64.62	31.50	0.02
2004	0.55	76.54	898.24	0.09	344.31	2078.39	9.80	114.06	2.73	6.14	29.11	3.31	18.74	34.72	0.00
2005	1.07	53.07	573.85	0.04	333.67	2705.77	27.21	118.43	2.96	2.07	20.01	4.14	38.60	32.00	0.00
2006	7.14	73.99	277.12	0.08	495.06	2930.04	36.79	130.31	5.09	1.23	21.90	3.68	59.03	34.78	0.00
2007	4.30	49.60	102.38	0.15	462.20	3193.12	31.77	139.51	2.76	1.45	47.67	4.66	80.64	29.86	0.00
2008	5.34	34.26	114.64	0.20	197.94	3902.60	35.04	174.66	2.85	1.10	93.26	5.55	83.67	35.06	0.01
2009	8.67	38.03	269.78	0.25	610.88	4635.90	42.10	237.52	2.75	2.99	52.79	6.82	74.98	59.70	0.00
2010	158.43	47.93	365.55	0.22	576.41	5706.23	68.44	264.45	3.62	5.89	90.26	5.88	54.21	74.54	0.01
2011	175.79	59.81	309.05	0.25	502.75	5469.43	79.00	323.50	3.00	5.45	136.67	8.31	42.11	90.60	0.01
2012	520.86	246.16	631.24	0.34	713.84	6230.16	74.23	328.65	2.95	10.52	135.92	13.29	52.19	114.58	0.00
2013	326.75	332.80	796.41	0.49	738.94	6786.67	110.60	315.69	2.40	33.36	140.00	25.89	58.43	159.22	0.00
2014	260.13	836.23	854.64	0.58	865.13	7755.23	86.30	387.57	2.34	37.68	138.36	28.32	46.90	181.30	0.00
2015	473.17	1407.96	1389.45	0.66	937.66	8759.60	100.19	434.06	2.95	54.43	159.31	22.37	40.86	161.06	0.00

Source: [2].

Table S16. The major import trading partners in 2015 (Unit: 10^4 t).

**Country (region)**	**Import volume**	**Proportion**
Brazil	4043.14	29.01%
America	3928.05	28.18%
Argentina	970.20	6.96%
Thailand	902.45	6.47%
Australian	832.86	5.98%
Canada	832.30	5.97%
Vietnam	497.72	3.57%
Ukraine	468.06	3.36%
French	463.58	3.33%
Others	1000.39	7.18%

Source: [2].

Table S17. Carbon emission factors (CEFs) of China's trading partners of agricultural products provided by FAO (Unit: t of CO_2-eq/t product).

Country (region)	Corn	Rice	Sugar	Wheat	Fruit	Oil crops	Beans	Tubers	Vegetables	Milk	Beef	Pork	Mutton	Poultry	Eggs
Afghanistan	0.29	1.98	0.03	0.31	0.39	0.44	0.17	0.07	0.18	8.79	8.77	-	20.30	1.95	5.49
Bahrain	-	-	-	-	5.18	-	0.12	0.97	1.04	1.47	14.53	-	17.47	0.98	0.87
Bangladesh	0.18	1.50	0.06	0.49	0.60	0.60	0.32	0.14	0.44	18.36	22.36	-	47.26	2.84	7.94
Bhutan	0.24	1.88	0.04	0.36	0.42	0.38	0.13	0.09	0.44	14.65	18.57	5.40	32.53	2.18	5.29
Brunei Darussalam	-	30.93	-	-	3.70	2.90	-	1.04	6.20	12.97	20.18	13.72	18.30	2.21	3.03
Myanmar	0.11	1.54	0.01	0.22	0.35	0.22	0.08	0.05	0.08	11.24	20.38	4.89	33.79	1.56	1.96
Cambodia	0.12	2.33	0.04	-	0.41	0.30	0.15	0.04	0.25	35.87	25.47	9.60	-	1.99	4.77
Cyprus	-	-	-	0.94	1.40	1.80	0.70	0.18	0.21	0.79	11.75	3.40	16.31	0.98	0.71
Democratic People's Republic of Korea	0.08	1.59	-	0.11	0.14	0.20	0.07	0.03	0.06	2.07	20.38	4.44	21.65	1.42	1.47
China, Hong Kong SAR	-	-	-	-	1.13	-	-	0.48	0.42	1.55	3.05	2.96	-	1.56	2.48
India	0.52	2.05	0.04	0.37	0.46	0.78	0.43	0.09	0.24	2.99	15.40	10.08	27.32	1.66	1.10
Indonesia	0.23	1.97	0.04	-	0.21	0.07	0.24	0.10	0.33	3.19	13.55	7.79	33.08	2.70	2.65
Iran (Islamic Republic of)	0.26	3.07	0.09	0.96	0.79	0.74	0.89	0.13	0.24	2.23	17.72	-	18.52	1.65	1.14
Iraq	0.58	4.05	0.11	0.66	1.15	0.50	0.41	0.19	0.33	4.48	8.26	-	15.57	1.46	1.18
Israel	0.15	-	-	1.93	0.88	1.12	0.25	0.21	0.46	0.27	4.98	4.61	14.15	1.30	0.79

Japan	1.18	2.44	0.10	0.96	0.88	1.87	0.52	0.21	0.33	0.65	7.26	3.21	19.24	1.04	14.95
Jordan	0.23	-	-	5.84	1.72	1.69	1.12	0.25	0.37	0.43	8.72	-	25.95	1.46	0.37
Kuwait	0.44	-	-	3.80	7.49	4.98	-	0.55	0.53	0.54	10.90	-	18.32	0.98	1.58
Lao People's Democratic Republic	0.11	1.28	0.04	-	0.29	0.27	0.14	0.08	0.27	28.40	24.45	14.11	-	2.48	2.24
Lebanon	0.24	-	0.08	0.32	0.32	0.45	0.15	0.07	0.10	0.70	7.93	7.92	14.68	0.61	0.54
China, Macao SAR	-	-	-	-	-	-	-	-	-	-	-	-	-	1.74	2.65
Malaysia	0.22	2.47	0.04	-	0.56	0.05	-	0.11	0.19	13.77	26.95	7.82	20.80	1.17	1.70
Maldives	0.02	-	-	-	0.01	-	0.01	0.01	0.04	-	-	-	-	-	-
Mongolia	-	-	-	10.26	159.17	9.92	4.29	2.48	4.41	9.94	26.93	7.36	17.65	1.11	2.40
Nepal	0.26	1.76	0.03	0.30	0.27	0.44	0.19	0.08	0.14	8.48	18.57	7.84	36.08	1.88	2.45
Oman	-	-	0.51	1.75	3.10	-	-	0.33	1.06	5.33	-	-	10.48	1.83	0.62
Pakistan	0.39	2.41	0.06	0.52	0.85	0.90	0.55	0.12	0.33	3.06	8.11	-	22.00	1.71	2.41
Occupied Palestinian Territory	-	-	-	0.39	0.25	0.38	0.16	0.02	0.03	0.58	8.51	-	9.89	0.86	0.56
Philippines	0.15	3.35	0.01	-	0.13	0.11	0.13	0.08	0.13	2.34	13.02	6.44	25.45	1.78	2.55
Qatar	1.45	-	-	12.46	15.46	-	-	4.59	6.41	2.16	14.53	-	20.96	1.05	0.63
Saudi Arabia	0.92	-	-	0.89	3.74	1.18	0.48	0.36	0.66	0.30	8.72	-	15.57	1.46	0.83
Singapore	-	-	-	-	27.84	3.08	-	2.66	4.10	-	-	7.76	-	1.00	1.82
Republic of Korea	0.89	2.69	-	1.25	1.19	2.86	0.96	0.36	0.37	0.50	9.34	2.91	23.32	1.92	1.11
Sri Lanka	0.22	1.32	0.02	-	0.38	0.15	0.13	0.08	0.18	5.85	11.60	5.56	16.54	1.71	1.42

Syrian Arab Republic	0.20	-	0.04	0.35	0.38	0.49	0.23	0.08	0.13	1.09	11.62	-	7.78	1.06	0.89
Thailand	0.19	2.63	0.02	0.78	0.43	0.08	0.23	0.06	0.32	1.97	10.08	3.05	24.49	1.58	1.73
Turkey	0.15	1.59	0.04	0.43	0.38	0.39	0.21	0.06	0.14	1.73	14.11	3.50	20.30	0.86	0.81
United Arab Emirates	0.10	-	-	0.88	3.03	-	-	0.18	0.27	2.86	6.97	-	17.47	1.22	0.77
Yemen	2.31	-	-	1.90	1.56	2.18	0.52	0.48	0.92	4.79	19.16	-	30.83	1.61	1.28
Viet Nam	0.21	1.59	0.03	-	0.35	0.26	0.30	0.10	0.23	2.36	17.76	5.38	-	1.32	2.98
China	0.36	1.75	0.06	0.41	0.89	0.63	0.33	0.19	0.26	1.65	21.71	2.89	20.82	1.13	1.14
China, mainland	0.36	1.75	0.06	0.41	0.90	0.63	0.34	0.20	0.26	1.66	21.71	2.90	20.82	1.13	1.13
China, Taiwan Province of	0.22	1.64	0.05	0.32	0.46	0.25	0.17	0.12	0.25	0.78	15.51	2.26	-	0.94	1.20
Timor-Leste	0.15	2.20	-	-	0.14	0.27	0.06	0.11	0.29	-	-	10.71	32.77	2.49	1.59
Kazakhstan	0.07	2.31	0.04	0.28	0.34	0.26	0.08	0.03	0.04	2.14	19.59	3.42	18.45	1.04	0.87
Kyrgyzstan	0.13	2.91	0.16	0.31	0.93	0.37	0.13	0.09	0.14	2.39	17.45	3.25	15.54	1.13	1.57
Tajikistan	0.11	2.37	-	0.35	1.50	1.21	0.10	0.08	0.13	7.78	17.29	5.45	21.65	1.82	2.20
Turkmenistan	1.36	6.03	0.31	0.62	0.56	2.54	0.25	0.45	0.24	2.36	16.98	3.92	21.65	1.24	0.70
Uzbekistan	0.39	2.81	-	0.61	1.60	2.47	0.38	0.20	0.27	2.85	16.98	3.17	17.09	1.11	1.24
Algeria	0.18	7.09	-	0.39	0.47	0.29	0.17	0.04	0.12	1.37	8.90	11.02	18.37	1.26	1.73
Angola	4.46	30.01	0.17	3.05	0.92	1.95	2.28	0.39	2.53	5.89	10.20	6.78	20.82	1.40	1.39
Benin	0.44	0.92	0.04	-	0.34	0.27	0.15	0.07	0.36	21.73	15.76	17.59	31.54	1.57	1.61
Botswana	36.00	-	-	-	14.07	7.29	11.11	1.51	3.97	8.07	8.67	12.97	22.31	1.83	1.36
Burundi	0.41	1.07	0.02	0.47	0.36	0.12	0.12	0.10	0.13	8.23	8.67	5.48	26.03	1.40	1.93
Cameroon	0.32	1.51	0.14	0.51	0.24	0.19	0.17	0.11	0.60	4.91	11.94	14.70	26.03	1.57	2.10

Cabo Verde	2.83	-	0.09	-	0.24	0.31	1.38	0.12	0.10	4.48	12.83	8.82	19.53	1.86	1.40
Central African Republic	7.53	14.50	1.99	-	7.41	4.91	1.53	2.96	3.17	10.79	11.24	15.47	20.82	1.47	1.82
Chad	1.65	4.40	0.04	0.71	2.34	1.51	0.66	0.41	0.57	10.46	14.44	17.66	17.72	1.79	2.02
Comoros	0.10	7.84	-	-	0.19	0.14	0.06	0.08	0.10	5.65	15.76	-	20.81	1.57	1.83
Congo	2.02	11.07	0.10	-	1.19	0.83	0.55	0.30	0.56	5.65	11.07	9.30	31.24	1.26	2.14
Djibouti	9.46	-	-	-	-	-	10.78	-	5.70	8.07	15.76	-	30.32	-	-
Egypt	0.29	1.68	0.04	0.37	0.51	0.59	0.20	0.15	0.27	1.55	5.48	18.00	8.42	0.91	0.79
Equatorial Guinea	-	-	-	-	0.03	0.01	-	0.02	-	-	-	9.80	28.40	1.56	1.16
Ethiopia	0.65	4.10	0.02	0.80	0.80	0.96	0.31	0.35	1.15	9.25	15.87	8.82	31.24	1.57	1.66
Gabon	0.47	1.54	0.03	-	0.70	0.40	0.24	0.23	0.55	11.30	13.13	15.75	26.03	1.57	1.39
Gambia	0.47	9.09	-	-	0.52	0.39	0.67	0.29	0.25	16.14	14.44	12.31	28.67	1.26	1.72
Ghana	0.29	1.51	0.04	-	0.20	0.20	1.41	0.07	0.18	21.73	13.87	10.50	21.03	1.71	2.75
Guinea	0.66	3.17	0.03	-	0.93	0.50	0.25	0.21	0.86	15.27	18.56	10.30	26.03	1.26	2.08
Guinea-Bissau	0.70	2.14	0.06	-	0.77	0.27	0.44	0.17	0.41	16.62	15.76	11.02	32.85	1.80	1.52
Côte d'Ivoire	0.14	0.97	0.01	-	0.31	0.05	0.08	0.07	0.21	20.14	16.03	13.48	21.90	1.77	1.89
Kenya	1.28	3.77	0.04	1.02	0.43	2.04	0.99	0.19	0.32	4.84	7.38	5.38	21.25	1.02	1.48
Liberia	-	0.83	0.04	-	0.16	0.02	0.08	0.04	0.12	21.74	13.86	11.02	31.24	1.57	1.46
Libya	1.06	-	-	3.41	2.20	2.38	0.44	0.21	0.55	2.68	9.59	-	20.82	0.97	1.15
Madagascar	0.94	3.42	0.10	0.59	1.34	1.27	0.36	0.34	0.68	9.27	13.59	6.30	26.03	1.57	1.69
Malawi	0.15	1.52	0.01	0.21	0.15	0.23	0.12	0.03	0.23	5.86	13.97	21.83	28.92	1.56	1.69
Mali	0.40	2.36	0.04	0.46	1.06	0.91	0.75	0.12	0.36	11.53	13.33	12.31	25.23	1.57	4.04
Mauritania	7.38	5.40	-	2.14	8.02	4.52	2.70	3.48	5.20	8.07	14.44	-	21.03	1.57	2.14

Mauritius	0.12	-	0.03	-	0.29	0.38	-	0.08	0.22	2.58	-	6.36	19.78	1.14	0.82
Morocco	1.14	1.91	0.03	0.50	0.49	0.65	0.38	0.09	0.12	2.28	8.95	8.82	21.84	1.06	1.29
Mozambique	1.10	5.68	0.04	0.90	0.86	1.91	0.86	0.39	0.72	16.62	11.55	7.35	26.03	1.40	1.50
Namibia	4.21	-	-	1.37	11.13	8.33	1.90	1.95	3.46	5.65	7.02	8.02	17.35	1.57	2.27
Niger	0.37	2.90	0.01	0.21	0.28	0.36	0.41	0.03	0.09	8.56	16.20	10.95	19.71	1.57	2.41
Nigeria	0.16	2.39	0.04	0.21	0.28	0.16	0.09	0.06	0.15	11.77	13.33	9.80	28.67	1.26	1.60
Réunion	0.15	2.44	0.03	-	0.75	0.73	0.34	0.15	0.59	3.01	7.22	4.90	26.45	1.23	0.66
Rwanda	0.23	0.64	0.03	0.26	0.24	0.49	0.12	0.08	0.15	5.45	16.67	10.50	26.03	1.39	2.40
Sao Tome and Principe	0.07	-	-	-	0.05	0.03	-	0.02	0.03	16.62	9.84	8.94	31.20	1.57	1.54
Senegal	0.66	1.48	0.02	-	0.71	0.66	0.69	0.18	0.26	13.53	11.70	12.04	25.03	1.08	1.91
Seychelles	-	-	-	-	0.19	0.23	-	0.09	0.45	5.12	7.64	5.90	-	1.26	0.72
Sierra Leone	0.35	1.72	0.01	-	0.28	0.09	0.11	0.07	0.14	11.30	19.26	7.59	28.67	1.40	3.17
Somalia	2.79	5.29	0.25	12.33	2.29	2.55	3.85	0.92	3.14	7.57	15.76	8.82	24.26	1.57	2.70
South Africa	0.52	6.20	0.10	0.87	0.60	1.03	0.48	0.18	0.44	0.91	6.46	3.39	13.12	0.88	0.66
Sudan (former)	1.38	3.36	0.05	1.57	1.00	2.22	0.98	0.60	0.54	7.72	14.28	-	25.03	1.26	1.43
United Republic of Tanzania	0.85	4.78	0.02	1.75	0.98	1.02	0.27	0.39	0.53	11.98	16.02	11.02	26.03	1.38	2.80
Togo	0.37	0.65	-	-	0.31	0.25	0.31	0.11	0.28	12.56	13.87	15.75	28.67	1.57	1.46
Tunisia	-	-	-	0.27	0.48	1.01	0.14	0.07	0.08	1.62	10.20	9.75	24.21	0.93	1.64
Uganda	0.29	1.86	0.02	0.44	0.63	0.51	0.34	0.16	0.56	8.07	11.59	7.35	22.31	0.97	1.46
Democratic Republic of the Congo	1.92	7.00	0.07	1.19	1.25	0.69	0.82	0.33	0.84	3.72	11.12	1-	30.93	2.08	2.34

Burkina Faso	0.54	4.57	0.02	-	0.67	0.85	0.44	0.16	0.29	24.94	15.34	20.52	35.04	1.57	2.08
Zambia	2.11	9.47	0.09	0.79	4.41	4.25	2.44	1.48	2.08	9.42	10.83	10.02	22.31	1.26	1.62
Zimbabwe	3.13	3.04	0.05	0.51	1.42	2.35	0.69	0.54	0.78	6.57	7.70	8.02	22.31	1.09	1.52
Lesotho	3.83	-	-	4.70	2.21	-	0.90	0.18	0.86	11.30	-	-	30.93	1.02	0.99
Swaziland	1.61	2.19	0.04	1.20	1.10	2.62	0.97	0.65	0.92	9.75	7.65	8.82	17.35	1.26	1.46
Eritrea	1.57	-	-	1.31	0.88	3.16	0.57	0.61	1.34	18.84	17.33	-	31.54	1.48	1.82
Belgium	0.31	-	0.10	0.40	0.57	0.81	0.18	0.15	0.35	1.57	14.53	4.51	0.51	1.82	16.22
Denmark	-	-	0.08	0.25	0.85	0.26	0.16	0.08	0.24	1.03	14.01	6.64	25.17	2.30	12.31
United Kingdom	-	-	0.08	0.34	0.91	0.40	0.20	0.10	0.37	1.19	10.82	5.55	25.55	1.91	19.63
Germany	0.18	-	0.06	0.23	0.55	0.21	0.16	0.07	0.17	1.27	11.58	4.73	24.45	2.44	14.57
France	0.17	3.71	0.04	0.22	0.78	0.25	0.09	0.06	0.22	1.48	13.46	5.08	32.97	2.27	17.30
Ireland	-	-	-	1.64	3.15	1.74	0.69	0.83	1.04	1.85	11.33	5.45	25.30	2.74	24.98
Italy	0.17	3.09	0.06	0.43	0.52	0.44	0.22	0.11	0.17	1.57	11.25	4.09	60.83	1.72	22.19
Luxembourg	0.63	-	-	0.59	1.37	0.49	0.28	0.20	0.28	1.26	10.38	6.17	27.16	1.67	15.72
Netherlands	1.18	-	0.44	1.72	2.22	2.87	1.16	0.60	0.87	1.21	18.71	5.22	24.10	1.79	14.40
Greece	0.14	2.93	0.05	0.55	0.51	0.49	0.21	0.10	0.15	2.52	17.79	9.98	47.76	1.97	30.56
Portugal	0.22	3.68	0.04	0.89	1.50	1.02	0.56	0.18	0.15	1.27	16.66	9.55	49.18	2.15	15.91
Spain	0.09	2.50	-	0.34	0.51	0.30	0.27	0.05	0.08	1.31	14.42	6.20	47.31	1.62	16.92
Albania	0.32	-	0.19	0.45	1.17	0.78	0.40	0.14	0.24	2.77	34.35	4.46	58.88	3.87	34.83
Austria	0.13	-	0.04	0.29	0.40	0.32	0.17	0.07		1.46	11.23	4.58	22.61	2.05	16.68
Bulgaria	0.14	1.67	-	0.21	1.30	0.16	0.15	0.07	0.12	2.05	25.15	3.95	56.37	1.83	22.07
Finland	-	-	0.14	0.60	4.37	0.70	0.25	0.15	0.26	1.10	12.07	5.04	27.77	1.60	15.06
Hungary	0.12	2.63	0.03	0.20	0.39	0.16	0.12	0.05	0.12	1.29	14.41	3.14	50.47	1.67	23.53
Iceland	-	-	-	-	-	-	-	84.78	17.69	1.86	19.61	5.81	31.16	1.89	18.35
Liechtenstein	-	-	-	-	-	-	-	-	-	1.99	-	-	-	-	-

Malta	-	-	-	0.23	1.29	1.13	0.11	0.13	0.23	1.78	15.71	8.69	30.93	1.77	17.66
Norway	-	-	-	2.09	5.40	2.22	0.61	0.51	0.87	1.42	13.39	5.42	24.93	-	18.35
Poland	0.23	-	0.06	0.35	0.74	0.23	0.17	0.13	0.16	1.46	17.95	3.38	55.20	1.74	22.45
Romania	0.16	1.97	0.03	0.22	0.36	0.17	0.14	0.06	0.11	2.02	22.90	3.54	49.99	1.89	41.67
Sweden	-	-	0.06	0.28	2.15	0.29	0.15	0.09	0.33	1.05	11.55	5.01	25.81	2.02	13.90
Switzerland	0.38	-	0.10	0.67	0.87	0.65	0.28	0.15	0.48	1.30	16.65	5.04	22.81	2.27	14.79
Estonia	-	-	-	0.96	12.61	0.86	0.50	0.33	0.38	1.01	17.62	3.79	27.59	1.73	15.24
Latvia	-	-	-	0.49	2.92	0.41	0.23	0.18	0.29	1.44	20.61	3.80	23.05	1.81	12.44
Lithuania	0.23	-	0.05	0.24	1.35	0.23	0.15	0.12	0.13	1.47	16.86	3.60	34.87	1.73	21.04
Georgia	0.49	-	-	1.10	1.52	1.79	0.16	0.17	0.40	4.93	42.57	6.54	18.35	1.22	1.11
Armenia	0.14	-	0.10	0.36	0.37	-	0.08	0.07	0.07	2.36	27.02	2.74	19.80	1.49	0.79
Azerbaijan	0.26	5.11	0.11	0.49	0.87	0.62	0.17	0.15	0.27	4.30	27.36	4.29	22.24	1.31	1.85
Belarus	0.45	-	0.10	0.62	1.41	0.67	0.23	0.20	0.24	1.55	21.76	3.90	26.49	2.36	19.80
Republic of Moldova	0.10	-	0.03	0.12	0.28	0.10	0.07	0.05	0.09	2.08	28.71	3.52	33.12	2.01	23.81
Russian Federation	0.19	2.16	0.04	0.29	0.48	0.28	0.10	0.08	0.10	1.89	20.15	3.51	29.94	1.91	18.14
Ukraine	0.09	1.80	0.03	0.15	0.31	0.14	0.07	0.05	0.09	1.73	24.23	3.22	33.12	1.61	26.75
Slovenia	0.33	-	-	0.66	1.16	0.64	0.35	0.20	0.41	1.21	13.26	3.16	37.32	1.81	17.60
Croatia	0.22	-	0.07	0.32	1.22	0.38	0.25	0.15	0.28	1.88	22.31	3.91	44.16	3.07	32.45
Czech Republic	0.13	-	0.04	0.22	0.81	0.21	0.14	0.07	0.18	0.99	13.05	3.30	34.19	2.13	18.45
Slovakia	0.11	-	0.03	0.18	0.64	0.16	0.12	0.07	0.20	1.21	14.72	3.10	62.34	1.91	24.36
The former Yugoslav	0.14	1.74	0.05	0.22	0.33	0.20	0.09	0.08	0.14	2.26	27.58	3.00	30.45	3.69	27.46

Republic of Macedonia															
Bosnia and Herzegovina	0.12	-	-	0.16	1.08	0.24	0.09	0.09	0.31	2.57	23.32	4.35	32.91	2.11	45.93
Faroe Islands	-	-	-	-	-	-	-	130.96	-	-	-	-	46.88	-	-
Serbia	0.13	-	0.03	0.20	0.76	0.18	0.07	0.11	0.26	2.41	16.72	6.25	27.46	1.78	39.74
Montenegro	0.30	-	-	0.31	1.27	1.37	0.13	0.13	0.17	3.30	21.44	2.57	0.53	2.09	36.34
Antigua and Barbuda	1.29	-	-	-	2.02	35.66	-	1.09	1.56	4.58	16.45	5.94	26.20	1.74	2.83
Argentina	0.17	1.75	0.03	0.36	0.32	0.51	0.23	0.08	0.17	0.89	14.59	3.18	30.53	0.79	0.92
Bahamas	0.20	-	0.10	-	0.24	-	0.50	0.53	0.68	4.40	-	5.50	22.23	1.71	1.45
Barbados	0.34	-	0.03	-	0.57	0.32	0.14	0.11	0.26	2.45	15.93	3.45	8.98	-	-
Belize	0.37	0.93	0.06	-	0.31	0.55	0.31	0.17	0.34	4.55	15.05	4.40	15.80	-	-
Bolivia (Plurinational State of)	0.44	2.73	0.05	0.98	0.75	0.61	0.28	0.39	1.01	2.11	23.11	5.50	36.63	1.57	3.02
Brazil	0.35	1.37	0.04	0.64	0.40	0.50	0.42	0.16	0.18	3.40	12.90	2.95	19.46	1.15	1.92
Cayman Islands	-	-	-	-	2.38	-	-	0.89	0.97	-	-	-	-	-	-
Chile	0.27	2.94	0.08	0.72	0.89	0.54	0.55	0.25	0.36	1.87	12.69	2.75	22.51	0.72	0.62
Colombia	1.10	3.48	0.07	1.80	1.19	0.16	0.63	0.40	0.56	3.71	14.51	3.53	21.48	2.07	0.88
Dominica	0.30	-	0.04	-	0.22	0.14	0.18	0.07	0.12	4.53	17.06	4.59	22.26	2.08	2.91
Costa Rica	0.72	1.32	0.04	-	0.27	0.08	0.79	0.20	0.17	3.24	13.29	3.67	20.78	1.14	0.74
Cuba	0.74	4.77	0.07	-	0.69	0.52	0.41	0.30	0.38	2.96	18.71	5.14	20.76	1.89	1.27
Dominican Republic	1.35	3.49	0.08	-	0.46	0.33	0.53	0.43	0.47	3.02	15.66	4.02	25.95	1.68	0.86

Ecuador	0.45	2.45	0.03	1.16	0.23	0.12	0.99	0.36	1.23	0.86	15.05	3.06	22.24	0.97	0.73
French Guiana	5.05	10.98	1.45	-	2.76	1.35	-	1.54	0.89	7.43	18.42	5.07	26.65	2.02	2.30
Grenada	0.46	-	0.02	-	0.37	0.15	0.08	0.16	0.12	5.57	19.74	5.45	15.71	1.89	1.52
Guadeloupe	-	-	0.04	-	0.29	0.75	0.93	0.19	0.39	5.74	14.64	4.23	18.75	1.59	1.38
Guatemala	0.39	1.15	0.02	0.38	0.15	0.07	0.22	0.06	0.12	6.03	16.65	5.50	20.76	1.15	0.99
Guyana	1.72	3.83	0.11	-	3.63	0.98	1.19	0.80	1.45	4.06	20.99	6.17	31.44	1.88	2.70
Haiti	0.61	2.28	0.02	-	0.34	0.39	0.17	0.20	0.40	15.33	20.26	4.59	17.11	2.30	1.57
Honduras	0.58	0.80	0.02	1.78	0.24	0.06	0.40	0.20	0.12	3.37	25.57	2.76	20.75	1.47	1.19
Jamaica	5.45	5.15	0.30	-	3.04	1.90	1.73	0.72	1.48	5.43	11.89	3.74	14.74	1.25	2.15
Martinique	-	-	0.02	-	0.07	0.38	0.21	0.11	0.10	0.76	19.03	5.40	16.47	1.81	0.53
Mexico	0.52	1.81	0.05	0.35	0.63	0.48	0.46	0.11	0.31	0.98	14.91	3.67	15.81	1.21	1.05
Montserrat	0.87	-	-	-	4.56	15.89	-	3.23	7.71	5.87	17.06	5.51	20.37	1.78	2.56
Nicaragua	0.84	1.07	0.03	-	0.76	0.18	0.42	0.28	1.63	6.03	18.97	6.60	25.99	1.07	1.65
Panama	1.20	2.96	0.08	-	0.49	0.27	1.24	0.41	0.49	4.08	14.15	3.72	-	1.23	1.44
Paraguay	0.33	1.61	0.04	0.38	0.26	0.52	0.29	0.10	0.39	1.89	11.97	3.44	20.76	2.07	1.00
Peru	0.55	1.71	0.03	1.19	0.68	0.25	0.39	0.24	0.40	2.10	21.78	5.05	25.52	1.02	0.85
Puerto Rico	1.60	-	-	-	0.72	0.26	0.87	0.52	0.45	1.14	15.20	1.69	25.99	1.31	1.27
Saint Lucia	-	-	-	-	0.33	0.23	0.08	0.32	0.21	3.16	16.87	5.73	15.72	1.73	1.68
Saint Vincent and the Grenadines	0.01	-	0.02	-	0.11	0.09	0.05	0.08	0.07	3.26	16.97	4.59	23.96	2.07	1.43
El Salvador	0.30	0.53	0.02	-	0.36	0.17	0.32	0.14	0.16	2.23	18.56	6.14	20.73	1.52	1.02
Suriname	1.70	5.12	0.23	-	0.77	0.82	1.02	0.32	0.76	1.44	17.41	4.62	26.22	1.22	3.54
Trinidad and Tobago	0.90	9.57	-	-	2.48	0.59	0.57	0.69	1.56	12.01	19.16	6.78	15.73	1.26	1.86

Uruguay	0.83	2.11	0.11	0.78	0.80	1.34	0.73	0.36	0.49	1.67	14.74	3.77	23.41	2.07	1.47
Venezuela (Bolivarian Republic of)	0.89	3.80	0.11	12.07	1.27	0.83	1.15	0.41	0.51	3.30	13.43	4.92	21.10	1.06	0.85
British Virgin Islands	-	-	-	-	16.67	-	-	-	-	-	17.06	5.50	22.25	-	-
Saint Kitts and Nevis	-	-	-	-	0.94	0.50	0.37	0.58	0.51	-	-	12.02	29.66	1.83	2.63
Saint Pierre and Miquelon	-	-	-	-	-	-	-	-	5.45	-	-	-	-	2.29	117.66
Netherlands Antilles	-	-	-	-	-	-	-	-	-	3.97	20.43	4.59	31.14	2.02	1.98
Canada	0.10	-	0.03	0.31	0.41	0.24	0.12	0.05	0.11	1.30	9.81	7.57	22.73	1.66	17.07
United States of America	0.11	1.87	0.03	0.39	0.23	0.42	0.15	0.04	0.11	1.17	9.04	7.29	16.31	1.51	17.42
Greenland	-	-	-	-	-	-	-	-	-	-	-	-	26.44	-	-
Bermuda	-	-	-	-	0.39	-	-	0.10	0.29	3.79	19.72	15.77	-	2.67	28.34
Australia	0.29	1.88	0.05	1.08	0.71	0.75	0.36	0.08	0.17	1.27	13.94	11.89	24.19	1.63	25.15
Cook Islands	-	-	-	-	1.28	0.49	-	0.06	0.18	-	-	27.61	-	3.59	2.93
Fiji	0.58	2.78	0.06	-	0.99	0.42	0.90	0.27	0.34	3.78	18.30	18.41	20.62	1.91	2.63
Nauru	-	-	-	-	0.23	0.04	-	-	0.09	-	-	27.61	-	-	3.86
New Caledonia	0.86	-	-	4.52	6.50	0.70	0.92	1.03	4.79	11.86	19.94	9.81	18.44	3.64	1.62
Vanuatu	1.04	-	-	-	0.26	0.31	-	0.12	0.09	33.59	17.14	16.24	-	2.88	4.85
New Zealand	2.44	-	-	3.74	7.48	11.01	2.71	1.08	3.17	1.89	20.83	10.94	27.82	1.67	17.61

Papua New Guinea	0.52	6.46	0.14	-	1.20	0.23	1.26	0.56	0.60	71.71	23.19	20.71	20.73	3.20	6.47
Solomon Islands	-	4.12	-	-	0.03	0.01	0.02	0.01	0.01	11.12	18.80	20.70	-	3.59	4.85
Tonga	-	-	-	-	2.37	0.11	-	0.15	0.64	5.10	18.31	18.20	-	3.27	4.85
Samoa	-	-	0.08	-	0.38	0.10	-	0.13	0.92	7.65	22.76	23.60	-	3.58	3.99
Kiribati	-	-	-	-	0.03	0.01	-	0.01	0.01	-	-	20.71	-	3.38	8.01
Tuvalu	-	-	-	-	0.12	0.32	-	-	0.09	-	-	27.61	-	2.79	13.34
Micronesia (Federated States of)	0.07	4.69	-	-	0.13	0.04	-	0.02	0.02	-	-	27.61	-	4.03	8.62
French Polynesia	-	-	0.01	-	0.12	0.10	-	0.04	0.06	3.33	23.61	14.11	20.56	3.20	1.13
Wallis and Futuna Islands	-	-	0.03	-	0.13	0.41	-	0.03	0.19	4.81	19.81	16.56	-	3.53	5.72
Falkland Islands (Malvinas)	-	-	-	-	-	-	-	-	-	4.98	11.76	-	17.30	-	-
Guam	0.03	-	-	-	0.04	0.02	-	0.01	0.01	-	-	13.81	-	3.59	2.16
Niue	-	-	-	-	0.21	0.04	-	0.01	0.01	10.46	16.41	12.90	-	3.51	4.12
Tokelau	-	-	-	-	0.02	0.01	-	0.01	-	-	-	27.61	-	2.15	-
United States Virgin Islands	-	-	-	-	-	-	-	-	-	1.49	14.06	3.87	22.22	-	-
Western Sahara	-	-	-	-	-	-	-	-	-	-	-	-	31.54	-	-

American Samoa	-	-	0.24	-	0.09	0.04	-	0.03	0.05	8.88	22.81	16.56	-	3.60	4.84

Source: [3].

References

1. Hawkins, J.; Ma, C.; Schilizzi, S., Zhang, F. Apples to kangaroos: A framework for developing internationally comparable carbon emission factors for crop and livestock products. *J. Clean Prod.* 2016,139, 460-472. https://doi.org/10.1016/j.jclepro.2016.08.060.
2. CCSY *1996-2016. China Customs Statistics Yearbooks 1996-2016*. China Customs Press: Beijing.
3. Food and Agricultural Organization of the United Nations. Food and agriculture data. 2022. Available online: http://www.fao.org/faostat/en/. (accessed on 25 February 2022).
